# Overall Survival Differences in Young Black Colorectal Cancer Patients: a Report from the National Cancer Database

**DOI:** 10.7150/jca.86634

**Published:** 2023-09-25

**Authors:** Macelyn Batten, Rupak Mukherjee, Thomas S. Walter, William P. Lancaster

**Affiliations:** 1 Division of Hepatobiliary Surgery, Department of Surgery, Medical University of South Carolina, Charleston, SC, United States.; 2 Division of Cardiothoracic Surgery, Department of Surgery, Medical University of South Carolina, Charleston, SC, United States.

**Keywords:** racial disparities, colorectal cancer, young onset

## Abstract

**Objectives:** Black patients have the highest overall incidence rate of early onset colorectal cancer, with many of these patients presenting with more aggressive disease at diagnosis, ultimately leading to decreased overall survival. We aimed to (1) evaluate how race and age affected overall survival in colorectal cancer patients, and (2) determine the different demographic and clinical covariables that may influence survival in younger individuals.

**Methods:** The 2017 National Cancer Database (NCDB) was used to identify all patients that had colorectal cancer between 2004-2017. These patients were then divided into groups according to age (<45 and ≥45 years old) and race (white and black). Overall survival (OS) between white and black groups according to age was compared. Initial testing of survivor functions between groups revealed violations of the proportional hazards assumption. Accordingly, we used parametric maximum likelihood analyses fitting the survivor functions to Weibull distributions. Logistic regression analysis was used to determine univariate and multivariate relationships between the covariates and race for younger subjects. Propensity score matching analysis was also used to control for differences in the demographic or clinical variables between the young black versus white subgroups.

**Results:** Out of 1.4 million potential cases initially identified, 207,823 unique cases were deemed eligible for evaluation based on study criteria. Black patients in the study population were more likely to be female, have medical comorbidities, and come from areas with lower average income and baseline education. OS was lower in older patients of both race categories when compared to the younger cohorts. Among patients older than 45 years, there were no significant differences in proportional hazard of death between black and white patients. However, among those younger than 45 years, younger black patients had significantly increased hazard of death. Regarding disease burden at diagnosis, pathologic characteristics and overall risk of death, there were no significant differences between black and white patients.

**Conclusions:** Overall survival in young black patients with colorectal cancer is significantly reduced when compared to young white patients, even when controlling for demographic and pathologic factors. This suggests that the outcome disparities between black and white patients are complex, and the underlying factors are not well understood.

## Introduction

In the United States, colorectal cancer (CRC) is projected to be the third leading cause of cancer related death in 2022 1. CRC disproportionately affects the black community; blacks have a higher probability of developing CRC, and there is evidence suggesting later stage disease at presentation in black patients 2, 3. Black patients have also been shown to have worse stage specific survival than non-Hispanic whites (NHW) 4. Additionally, blacks tend to be younger at presentation than other groups 5.

Early onset colorectal cancer (EOCRC) is CRC diagnosed in patients less than 50 years old. Since the 1970s, the incidence rate of EOCRC has increased for all groups; however, blacks have the highest overall incidence rate of EOCRC 5-8. Survival in black EOCRC patients is also reduced compared to NHW patients 4-9.

Relative to other groups, there is a higher prevalence of proximal colon cancers in black EOCRC patients 10, 11. Proximal colon cancers are associated with a lower five-year survival rate than distal colon cancers 12. EOCRC is also associated with more aggressive histology in blacks than in other groups 13, 14.

Taken together, there is an increased number of young black patients presenting with advanced and aggressive CRC that is less responsive to conventional therapy. Because of these trends, we hypothesize that there is a distinct and aggressive CRC phenotype that disproportionately affects young black patients and is associated with worse survival outcomes.

## Methods

### Data source, variables, and population

All data was collected from the National Cancer Database (NCDB) 2017 public use file (PUF). The NCDB is the largest cancer registry in the world and is estimated to include 70% of all cancer diagnoses in the US and Puerto Rico. Databases for colon cancer, rectal cancer, and rectosigmoid junction cancers were utilized. Accuracy of the data is maintained through audits and rigorous training of their data collectors, the Certified Tumor Registrars. Data in the PUF follows the privacy requirements of the Health Information Portability and Accountability Act and does not include patient or hospital identifiers. The Institutional Review Board at the Medical University of South Carolina determined this study was exempt, as it uses publicly available de-identified data.

The variables that were used for analysis included age, sex, race, Charlson-Deyo score, median income, education level, clinical stage of cancer (a composite of the T, M, and N staging system), tumor grade, and lymphovascular invasion. Given that the recommended age for initial colonoscopy screening is 45-years-old 6, age was dichotomized to <45 years and ≥45 years for the younger and older groups, respectively. Only patients that self-identified as white or black were included. The modified Charlson-Deyo score as defined by the Commission on Cancer (CoC) was dichotomized to none (0) and present (≥1). Patients were divided into quartiles for their median income and education level based on the zip code of their residence as determined in 2012. To minimize the influence of missing data on multivariate analysis, patients were excluded if they had any missing data for the clinical and pathologic variables described above.

### Outcomes and analysis

Our primary outcome was overall survival (OS). To minimize the risk of immortal bias, survival was indexed to initiation of treatment or surgical resection, whichever was earlier. Subjects were followed through the earliest of last contact, death, or a follow up period of 60 months (about 5 years). We used the Kaplan-Meier analysis to compare survival between white and black groups and between age groups. Initial testing of survivor functions between groups revealed violations of the proportional hazards assumption. Accordingly, we used parametric maximum likelihood analyses fitting the survivor functions to Weibull distributions. Logistic regression analysis was used to determine univariate and multivariate relationships between the covariates and race for younger subjects. Given the discrepancy in clinical parameters of young white patients versus young black patients, a propensity score matching analysis was also performed. Propensity score matching analyses were performed in two sets: one to propensity match demographic variables and a second to propensity match clinical variables. Both sets of propensity score matching analysis were performed using the nearest neighbor algorithm without replacement (1:1 match between groups) with calipers set to 0.2. All statistical analyses were performed using the Stata statistical software package (version 17.0, College Station, TX). A p-value less than 0.05 for all tests was considered to be statistically significant.

## Results

### Patient characteristics

**Figure 1:**
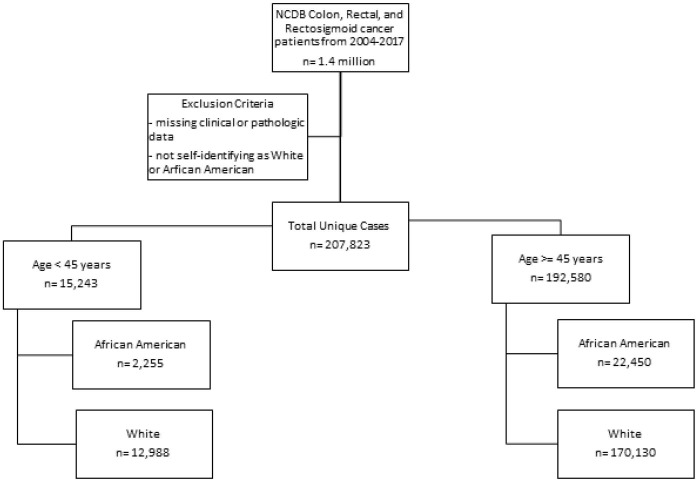
Consort diagram for sample sizes in each group

**Figure 2:**
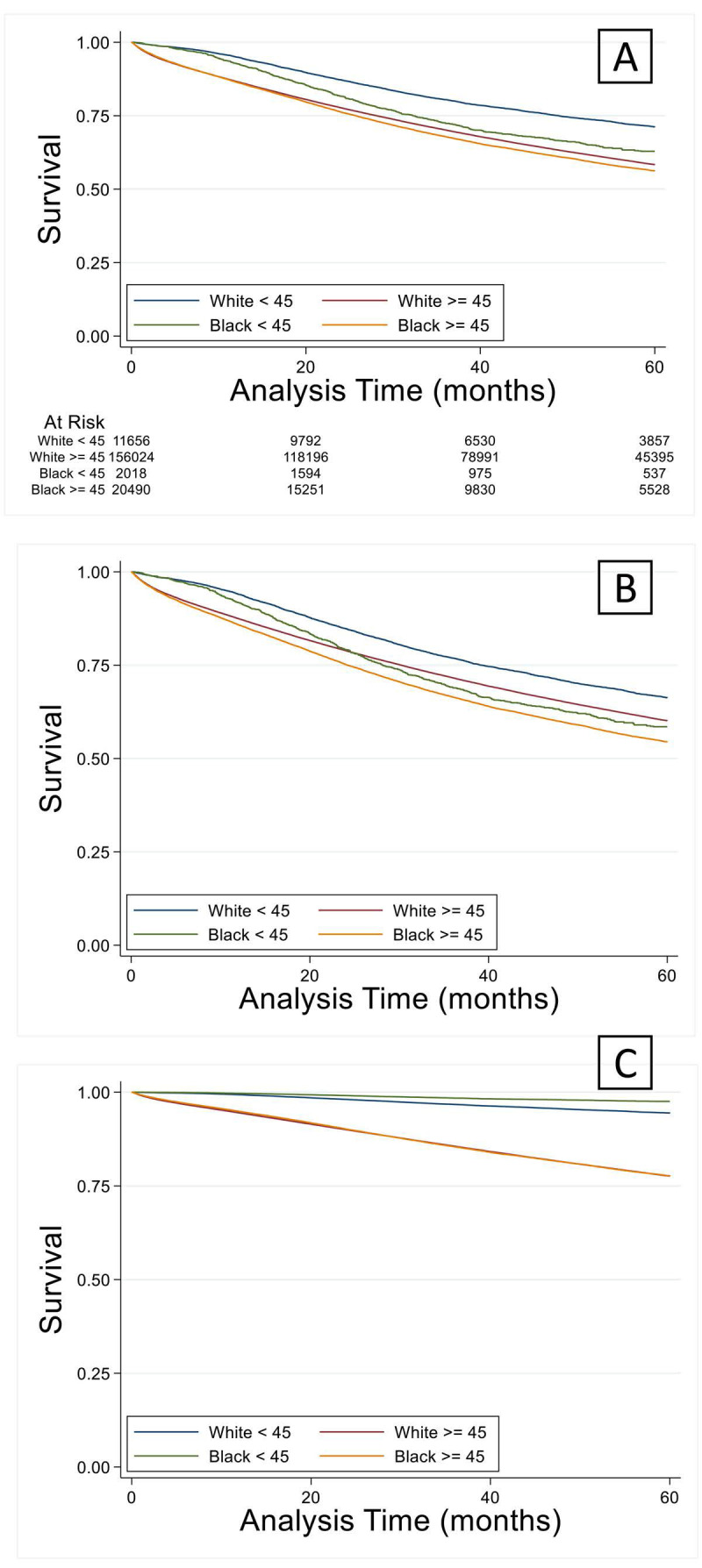
Overall survival by Kaplan-Meier analysis. (A) Overall survival was lower (p < 0.05) in both older groups compared to the race-matched younger cohorts. This finding remained true when controlling for demographic (B) or cancer staging factors (C).

A total of 1.4 million patients were identified of which 768,000 were excluded due to incomplete data. The number of unique complete cases included in the analysis was 207,823. Most patients were white and older than 45 years (n=170,130) (Figure 1). Among patients older than 45 years, black patients were more likely to have a lower income and less education when compared to white patients (Table 1a). Older black and white patients had a similar stage at presentation and similar tumor pathologic characteristics. Overall survival (OS) by Kaplan-Meier analysis is summarized in Figure 2. OS was lower in older patients of both race categories when compared to the younger cohorts. However, among patients older than 45 years, there were no significant differences in proportional hazard of death between black and white patients (Figure 2A), which persisted when controlling for demographic (Figure 2B) or pathologic variables (Figure 2C).

For patients younger than 45 years, subjects in the black group were significantly more likely to have comorbidities, have a lower income, and be less educated than white patients (Table 1b). A significantly higher number of black patients presented with stage 4 disease and had lymphovascular invasion present. In the younger patient cohort, univariate logistic analysis revealed all variables related to demographics and socioeconomic status were statistically different between white and black patients (Table 2). Young black patients were more likely to be female and have comorbidities present. In addition, they were significantly more likely to fall within the worst income quartile and the least educated quartile. However, clinical and pathologic characteristics were not significantly different between the young black and white populations. Patients were equally likely to present with stage 4 disease regardless of race or lymphovascular invasion. Degree of tumor differentiation were similar between races.

**Figure 3:**
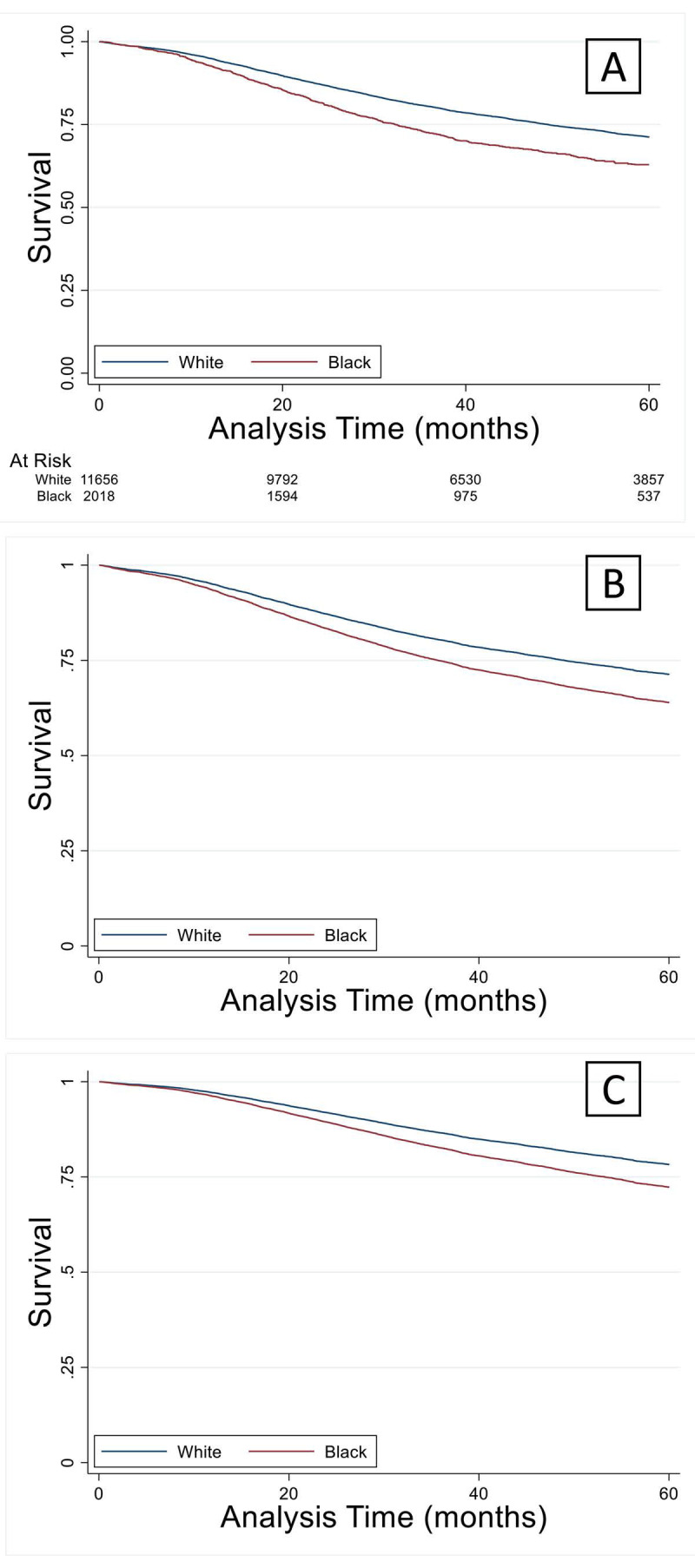
(A) Overall survival in the younger black group was significantly lower (p < 0.05) compared to their white counterparts and remained lower when controlled for demographics (B) or cancer staging factors (C).

Multivariable logistic analysis demonstrated similar results to univariable analysis (Table 3). Young black patients were more likely to be female with comorbidities. There were significant income and educational differences between black and white patients. Staging and pathologic variables were similar to the findings of univariable analysis. Younger black patients had significantly increased hazard of death compared to white counterparts (Figure 3A). These differences were not abrogated by controlling demographic or pathologic variables (Figures 3B and 3C).

**Figure 4:**
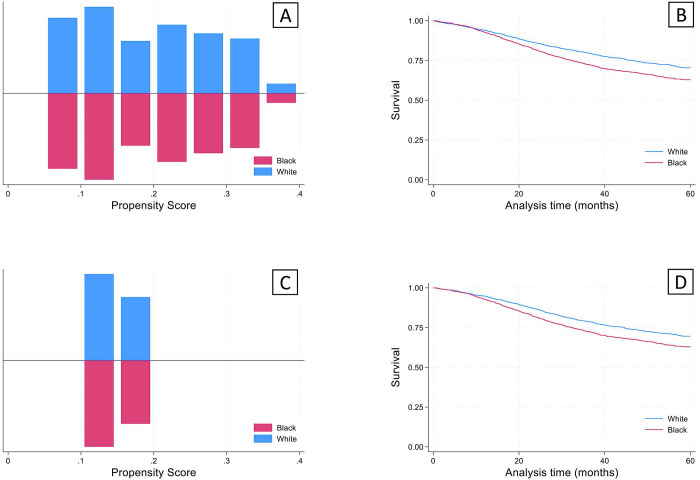
Propensity score matching analysis was performed for Social/Demographic factors (Sex, Comorbidities, Income, and Education) and cancer staging factors (Clinical stage, Lymphovascular invasion, and Grade) to achieve a 1:1 match using the nearest neighbor algorithm. A concordant group of young white subjects was identified for each of the analyses, giving a final sample size of 2,255 for each of the groups. (A) Results of propensity scores for matching on social/demographic factors. (B) Kaplan-Meier analysis revealed significantly lower survival (log-rank test) in the young black group compared to the young white group (p<0.05). (C) Propensity scores for matching on cancer staging factors. (B) Kaplan-Meier analysis revealed significantly lower survival (log-rank test) in the young black group compared to the young white group (p<0.05).

Given that there were significant differences in demographics and clinical characteristics between the young white and black groups, propensity score matching analysis was performed. The propensity scores after nearest neighbor matching created balanced 1:1 cohorts for both sets of analyses (Figure 4). After using propensity score matching within the young black and white groups for socioeconomic and clinical variables, a significant survival deficit remained in the young black group compared to the young white group (Figure 4).

## Discussion

In this paper, we chose to focus on young patients affected by colorectal cancer for many reasons. First, clinical observation suggests that a more aggressive colorectal cancer phenotype is more common in young black patients 4. Secondly, younger patients tend to be healthier and thus potential confounders can be limited in this population. We found that black patients across the study population were more likely to be female and have medical comorbidities. Black patients were also more likely to be from lower income and lower education areas. With these findings, young black patients had a significantly worse risk of death than young white patients. Regarding disease burden at diagnosis, pathologic characteristics and overall risk of death, there were no significant differences noted when comparing black and white patients.

Significant disparities exist in colorectal cancer between black patients and patients of other races. These disparities are not limited to patient outcomes. Disparities have been demonstrated in time to first treatment, treatment offered, and overall access to care 15. There is also data to suggest that black patients have a higher prevalence of a more aggressive phenotype than white patients. While all of these disparities have been reliably demonstrated, the reason for a survival disparity in colorectal cancer among black and white patients is not well understood 16, 17.

It has been suggested that socioeconomic factors are a major contributor to survival disparities in colorectal cancer. Yu et al reported a significant survival difference between black and white patients when income level is considered 18. Similarly, there is data to suggest that geographic region and access to care are an important mechanism underlying survival differences 19. However, contrary results have also been shown. For example, Katz et al found that in an equal access system, disparities observed in the general population disappear 20.

Important pathologic differences between black and white patients with colorectal cancer have also been demonstrated. Wallace and colleagues found that black patients had a higher incidence of more aggressive histology and right-sided colon cancers, both of which are associated with worse survival outcomes. They propose this as a possible explanation for the observed outcome disparities 21, 22. Additionally, colon cancers in black patients have been shown to display a more pronounced lymphocytic reaction at the time of diagnosis. While there was no difference in survival between black and white patients with high lymphocyte scores, there was a difference in survival noted among black patients with no lymphocytic reaction compared to white patients with no reaction 23.

Limitations of our study include those often encountered with usage of large databases like the NCDB. Selection bias, limited access to follow-up information as well as other patient reported complications may also be underreported with this databank. Additionally, the NCDB is a reporting system that is based upon information from hospitals not populations, so access to data representative of an entire population may not be accurately reflected. Other important clinical factors we could have considered when evaluating how social determinants of health contribute to overall survival in this population would be insurance status and treatment course. Health insurance coverage disparities have been shown to account for up to one half of the survival disparity in black versus white colorectal cancer patients 24. Also, treatment trends in young versus older patients with colorectal cancer have proven to be widely variable, which could also lead to discrepancies in survival for these populations 25.

When comparing unadjusted survival outcomes, black patients in both young and old groups had significantly worse survival. Adjusting for disease characteristics and socioeconomic variables eliminated the observed survival difference between races in patients older than 45. However, significant differences persisted in young patients after controlling for socioeconomic variables. This suggests that the outcome disparities between black and white patients are complex, and the underlying factors are not well understood.

## Disclaimer

The National Cancer Database (NCDB) is a joint project of the Commission on Cancer (CoC) of the American College of Surgeons and the American Cancer Society. The data used in the study are derived from a de-identified NCDB file. The American College of Surgeons and the Commission on Cancer have not verified and are not responsible for the analytic or statistical methodology employed, or the conclusions drawn from these data by the investigator.

## Figures and Tables

**Table 1a:** Patient Demographic and Clinical Characteristics of all White and Black Subjects

	All patients n=207,823	Young white n=12,988	Young black n=2,255	Old white n=170,130	Old black n=22,450	p-value
Age, (years; mean±SD)	65.6±13.7	38.8±5.8	38.7±5.8	68.1±11.8*	64.7±11.0*^+^	<0.001
Age, dichotomized (n, %)						<0.001
≤45 years	15,243 (7.3)	12,988	2,255			
>45 years	192,580 (92.7)			170,130	22,450	
Sex (n, %)						<0.001
Male	109,114 (52.5)	6,824 (52.5)	1,057 (46.9)	90,146(53.0)	11,087 (49.4)	
Female	98,709 (47.5)	6,164 (47.5)	1,198 (53.1)	79,984 (47.0)	11,363 (50.6)	
Race (n,%)						<0.001
White	183,118 (88.1)	12,988		170,130		
Black	24,705 (11.9)		2,255		22,450	
Charlson-Deyo Score (n, %)						<0.001
0	147,535 (71.0)	11,631 (89.6)	1,948 (86.4)	118,834 (69.9)	15,122 (67.4)	
1	42,317 (20.4)	1,159 (8.9)	245 (10.9)	35,818 (21.1)	5,095 (22.7)	
2	11,967 (5.8)	126 (1.0)	45 (2.0)	10,366 (6.1)	1,430 (6.4)	
3	6,004 (2.9)	72 (0.6)	17 (0.8)	5,112 (3.0)	803 (3.6)	
CDCC Present (n, %)						<0.001
No comorbidity	147,535 (71.0)	11,631 (89.6)	1,948 (86.4)	118,834 (69.9)	15,122 (67.4)	
Comorbidities present	60,288 (29.0)	1,357 (10.5)	307 (13.6)	51,296 (30.2)	7,328 (32.6)	
Median Income (n, %)						<0.001
Lowest quartile	37,556 (18.0)	1,811 (13.9)	817 (36.2)	25,203 (14.8)	9,725 (43.3)	
Second quartile	50,439 (24.3)	2,975 (22.9)	504 (22.4)	41,933 (24.7)	5,027 (22.4)	
Third quartile	55,496 (26.7)	3,543 (27.3)	500 (22.2)	47,203 (27.8)	4,250 (18.9)	
Highest quartile	64,332 (31.0)	4,659 (35.9)	434 (19.3)	55,791 (32.8)	3,448 (15.4)	
No High School Diploma (n, %)						<0.001
Highest quartile (>21%)	35,401 (17.0)	2,094 (16.1)	700 (31.0)	24,877 (14.6)	7,730 (34.4)	
Second quartile (13-21%)	55,974 (26.9)	3,194 (24.6)	733 (32.5)	43,911 (25.8)	8,136 (36.2)	
Third quartile (7-13%)	68,716 (33.1)	4,279 (33.0)	569 (25.2)	59,154 (34.8)	4,714 (21.0)	
Lowest quartile (<7%)	47,732 (23.0)	3,421 (26.3)	253 (11.2)	42,188 (24.8)	1,870 (8.3)	
Clinical stage (n, %)						<0.001
0	9,249 (4.5)	359 (2.8)	82 (3.6)	7,542 (4.4)	1,266 (5.6)	
1	66,752 (32.1)	3,211 (24.8)	551 (24.4)	56,064 (33.0)	6,916 (30.8)	
2	50,809 (24.4)	2,640 (20.3)	394 (17.5)	42,762 (25.1)	5,013 (22.3)	
3	40,913 (19.7)	3,553 (27.4)	540 (24.0)	32,618 (19.2)	4,202 (18.7)	
4	40,100 (19.3)	3,215 (24.8)	688 (30.5)	31,144 (18.3)	5,053 (22.5)	
Lymph vascular invasion (n, %)						<0.001
Not present	145,644 (70.1)	8,456 (65.1)	1,414 (62.7)	120,040 (70.6)	15,734 (70.1)	
Present / identified	62,179 (29.9)	4,532 (34.9)	841 (37.3)	50,090 (29.4)	6,716 (29.9)	
Grade/Differentiation (n, %)						<0.001
Well differentiated	26,794 (12.9)	1,833 (14.1)	363 (16.1)	21,388 (12.6)	3,210 (14.3)	
Moderately differentiated	141,176 (67.9)	8,642 (66.5)	1,416 (62.8)	115,595 (67.8)	15,523 (67.9)	
Poorly differentiated	33,968 (16.3)	2,143 (16.5)	408 (18.1)	28,170 (16.6)	3,247 (14.5)	
Undifferentiated	5,885 (2.8)	370 (2.9)	68 (3.0)	4,977 (2.9)	470 (2.1)	

**Table 1b:** Patient Demographic and Clinical Characteristics of Young White and Black Subjects

	All patients n=207,823	Young white n=12,988	Young black n=2,255	p-value
Age, (years; mean±SD)	65.6±13.7	38.8±5.8	38.7±5.8	<0.001
Age, dichotomized (n, %)				<0.001
≤45 years	15,243 (7.3)	12,988	2,255	
>45 years	192,580 (92.7)			
Sex (n, %)				<0.001
Male	109,114 (52.5)	6,824 (52.5)	1,057 (46.9)	
Female	98,709 (47.5)	6,164 (47.5)	1,198 (53.1)	
Race (n,%)				<0.001
White	183,118 (88.1)	12,988		
Black	24,705 (11.9)		2,255	
Charlson-Deyo Score (n, %)				<0.001
0	147,535 (71.0)	11,631 (89.6)	1,948 (86.4)	
1	42,317 (20.4)	1,159 (8.9)	245 (10.9)	
2	11,967 (5.8)	126 (1.0)	45 (2.0)	
3	6,004 (2.9)	72 (0.6)	17 (0.8)	
CDCC Present (n, %)				<0.001
No comorbidity	147,535 (71.0)	11,631 (89.6)	1,948 (86.4)	
Comorbidities present	60,288 (29.0)	1,357 (10.5)	307 (13.6)	
Median Income (n, %)				<0.001
Lowest quartile	37,556 (18.0)	1,811 (13.9)	817 (36.2)	
Second quartile	50,439 (24.3)	2,975 (22.9)	504 (22.4)	
Third quartile	55,496 (26.7)	3,543 (27.3)	500 (22.2)	
Highest quartile	64,332 (31.0)	4,659 (35.9)	434 (19.3)	
No High School Diploma (n, %)				<0.001
Highest quartile (>21%)	35,401 (17.0)	2,094 (16.1)	700 (31.0)	
Second quartile (13-21%)	55,974 (26.9)	3,194 (24.6)	733 (32.5)	
Third quartile (7-13%)	68,716 (33.1)	4,279 (33.0)	569 (25.2)	
Lowest quartile (<7%)	47,732 (23.0)	3,421 (26.3)	253 (11.2)	
Clinical stage (n, %)				<0.001
0	9,249 (4.5)	359 (2.8)	82 (3.6)	
1	66,752 (32.1)	3,211 (24.8)	551 (24.4)	
2	50,809 (24.4)	2,640 (20.3)	394 (17.5)	
3	40,913 (19.7)	3,553 (27.4)	540 (24.0)	
4	40,100 (19.3)	3,215 (24.8)	688 (30.5)	
Lymph vascular invasion (n, %)				<0.001
Not present	145,644 (70.1)	8,456 (65.1)	1,414 (62.7)	
Present / identified	62,179 (29.9)	4,532 (34.9)	841 (37.3)	
Grade/Differentiation (n, %)				<0.001
Well differentiated	26,794 (12.9)	1,833 (14.1)	363 (16.1)	
Moderately differentiated	141,176 (67.9)	8,642 (66.5)	1,416 (62.8)	
Poorly differentiated	33,968 (16.3)	2,143 (16.5)	408 (18.1)	
Undifferentiated	5,885 (2.8)	370 (2.9)	68 (3.0)	

**Table 2:** Univariate Logistic Analysis of Demographic and Clinical Characteristics Between Young White and Black Groups

	Odds Ratio	95% CI	p-value
Sex			
Male	base	--	
Female	1.25	[1.15-1.37]	<0.001
CDCC Present			
No comorbidity	base	--	
Comorbidities present	1.35	[1.18-1.54]	<0.001
Median Income			
Lowest quartile	base	--	
Second quartile	0.38	[0.33-0.43]	<0.001
Third quartile	0.31	[0.28-0.35]	<0.001
Highest quartile	0.21	[0.18-0.23]	<0.001
No High School Diploma			
Highest quartile (>21%)	base	--	
Second quartile (13-21%)	0.69	[0.61-0.77]	<0.001
Third quartile (7-13%)	0.40	[0.35-0.45]	<0.001
Lowest quartile (<7%)	0.22	[0.19-0.26]	<0.001
Clinical stage			
0	base	--	
1	0.75	[0.58-0.97]	0.027
2	0.65	[0.50-0.85]	0.001
3	0.67	[0.51-0.86]	0.002
4	0.94	[0.73-1.21]	0.614
Lymph vascular invasion			
Not present	base	--	
Present / identified	1.11	[1.01-1.22]	0.028
Grade/Differentiation			
Well differentiated	base	--	
Moderately differentiated	0.83	[0.73-0.94]	0.003
Poorly differentiated	0.96	[0.82-1.12]	0.617
Undifferentiated	0.93	[0.70-1.23]	0.604

**Table 3:** Multivariate Logistic Analysis of Demographic and Clinical Characteristics Between Young White and Black Groups

	Odds Ratio	95% CI	p-value
Sex			
Male	base	--	
Female	1.21	[1.11-1.33]	<0.001
CDCC Present			
No comorbidity	base	--	
Comorbidities present	1.21	[1.05-1.38]	0.008
Median Income			
Lowest quartile	base	--	
Second quartile	0.42	[0.37-0.48]	<0.001
Third quartile	0.40	[0.34-0.46]	<0.001
Highest quartile	0.36	[0.30-0.42]	<0.001
No High School Diploma			
Highest quartile (>21%)	base	--	
Second quartile (13-21%)	0.94	[0.83-1.07]	0.352
Third quartile (7-13%)	0.70	[0.60-0.81]	<0.001
Highest quartile (<7%)	0.42	[0.35-0.52]	<0.001
Clinical stage			
0	base	--	
1	0.76	[0.58-0.98]	0.038
2	0.64	[0.49-0.84]	0.001
3	0.69	[0.53-0.90]	0.006
4	0.92	[0.71-1.21]	0.564
Lymph vascular invasion			
Not present	base	--	
Present / identified	1.04	[0.94-1.16]	0.449
Grade/Differentiation			
Well differentiated	base	--	
Moderately differentiated	0.85	[0.75-0.98]	0.020
Poorly differentiated	0.96	[0.81-1.11]	0.609
Undifferentiated	0.91	[0.68-1.23]	0.549

## References

[1] Ashktorab H, Vilmenay K, Brim H, Laiyemo AO, Kibreab A, Nouraie M (2016). Colorectal Cancer in Young African Americans: Is It Time to Revisit Guidelines and Prevention?. Dig Dis Sci.

[2] DeSantis CE, Miller KD, Goding Sauer A, Jemal A, Siegel RL (2019). Cancer statistics for African Americans, 2019. CA Cancer J Clin.

[3] Mandelblatt J, Andrews H, Kao R, Wallace R, Kerner J (1996). The late-stage diagnosis of colorectal cancer: demographic and socioeconomic factors. Am J Public Health.

[4] Murphy CC, Wallace K, Sandler RS, Baron JA (2019). Racial Disparities in Incidence of Young-Onset Colorectal Cancer and Patient Survival. Gastroenterology.

[5] Rahman R, Schmaltz C, Jackson CS, Simoes EJ, Jackson-Thompson J, Ibdah JA (2015). Increased risk for colorectal cancer under age 50 in racial and ethnic minorities living in the United States. Cancer Med.

[6] Augustus GJ, Ellis NA (2018). Colorectal Cancer Disparity in African Americans: Risk Factors and Carcinogenic Mechanisms. Am J Pathol.

[7] Bailey CE, Hu CY, You YN, Bednarski BK, Rodriguez-Bigas MA, Skibber JM (2015). Increasing disparities in the age-related incidences of colon and rectal cancers in the United States, 1975-2010. JAMA Surg.

[8] Chang SH, Patel N, Du M, Liang PS (2022). Trends in Early-onset vs Late-onset Colorectal Cancer Incidence by Race/Ethnicity in the United States Cancer Statistics Database. Clin Gastroenterol Hepatol.

[9] Zaki TA, Liang PS, May FP, Murphy CC (2023). Racial and Ethnic Disparities in Early-Onset Colorectal Cancer Survival. Clin Gastroenterol Hepatol.

[10] Petrick JL, Barber LE, Warren Andersen S, Florio AA, Palmer JR, Rosenberg L (2021). Racial Disparities and Sex Differences in Early- and Late-Onset Colorectal Cancer Incidence, 2001-2018. Front Oncol.

[11] Shavers VL (2007). Racial/ethnic variation in the anatomic subsite location of in situ and invasive cancers of the colon. J Natl Med Assoc.

[12] Wong R (2010). Proximal tumors are associated with greater mortality in colon cancer. J Gen Intern Med.

[13] Lin J, Qiu M, Xu R, Dobs AS (2015). Comparison of survival and clinicopathologic features in colorectal cancer among African American, Caucasian, and Chinese patients treated in the United States: Results from the surveillance epidemiology and end results (SEER) database. Oncotarget.

[14] Xia C, Dong X, Li H, Cao M, Sun D, He S (2022). Cancer statistics in China and United States, 2022: profiles, trends, and determinants. Chin Med J (Engl).

[15] Hao S, Parikh AA, Snyder RA (2022). Racial Disparities in the Management of Locoregional Colorectal Cancer. Surg Oncol Clin N Am.

[16] Carethers JM, Doubeni CA (2020). Causes of Socioeconomic Disparities in Colorectal Cancer and Intervention Framework and Strategies. Gastroenterology.

[17] Dimou A, Syrigos KN, Saif MW (2009). Disparities in colorectal cancer in African-Americans vs Whites: before and after diagnosis. World J Gastroenterol.

[18] Yu XQ, Goldsbury D, Feletto E, Koh CE, Canfell K, O'Connell DL (2022). Socioeconomic disparities in colorectal cancer survival: contributions of prognostic factors in a large Australian cohort. J Cancer Res Clin Oncol.

[19] Carmichael H, Cowan M, McIntyre R, Velopulos C (2020). Disparities in colorectal cancer mortality for rural populations in the United States: Does screening matter?. Am J Surg.

[20] Lee S, Reha JL, Tzeng CW, Massarweh NN, Chang GJ, Hetz SP (2013). Race does not impact pancreatic cancer treatment and survival in an equal access federal health care system. Ann Surg Oncol.

[21] Wallace K, DeToma A, Lewin DN, Sun S, Rockey D, Britten CD (2017). Racial Differences in Stage IV Colorectal Cancer Survival in Younger and Older Patients. Clin Colorectal Cancer.

[22] Wallace K, Hill EG, Lewin DN, Williamson G, Oppenheimer S, Ford ME (2013). Racial disparities in advanced-stage colorectal cancer survival. Cancer Causes Control.

[23] Wallace K, Lewin DN, Sun S, Spiceland CM, Rockey DC, Alekseyenko AV (2018). Tumor-Infiltrating Lymphocytes and Colorectal Cancer Survival in African American and Caucasian Patients. Cancer Epidemiol Biomarkers Prev.

[24] Sineshaw HM, Ng K, Flanders WD, Brawley OW, Jemal A (2018). Factors That Contribute to Differences in Survival of Black vs White Patients With Colorectal Cancer. Gastroenterology.

[25] Galadima HI, Adunlin G, Hughes MS, Cropp CD, Lucero L, Akpinar-Elci M (2021). Racial disparities and treatment trends among young-onset colorectal cancer patients: An analysis of a hospital cancer registry. Cancer Epidemiol.

